# Digital radiography image quality evaluation using various phantoms and software

**DOI:** 10.1002/acm2.13823

**Published:** 2022-11-07

**Authors:** Ioannis A. Tsalafoutas, Shady AlKhazzam, Virginia Tsapaki, Huda AlNaemi, Mohammed Hassan Kharita

**Affiliations:** ^1^ Medical Physics Section OHS Department Hamad Medical Corporation Doha Qatar; ^2^ NAHU—Dosimetry and Medical Radiation Physics Section IAEA Vienna Austria; ^3^ Weill Cornell Medicine‐Qatar Doha Qatar

**Keywords:** detectability, digital radiography, image quality, MTF, QC phantoms, SDNR, SNR

## Abstract

**Purpose:**

To investigate the effect of the exposure parameters on image quality (IQ) metrics of phantom images, obtained automatically using software or from visual evaluation.

**Methods:**

Three commercial phantoms and a homemade phantom constructed according to the instructions given in the IAEA Human Health Series No. 39 publication were used, along with the respective software that estimate automatically various IQ metrics. Images with various exposure parameters were acquired in a digital radiography (DR) unit. For the commercial phantoms, visual evaluations were also performed. The IQ scores obtained were analyzed to investigate the effects of increasing incident air kerma (IAK), tube potential (kVp), additional filtration, and acquisition protocol on IQ.

**Results:**

The effects of the exposure parameters on the IQ metrics, determined with the commercial and the IAEA phantoms, were not the same. For example, clear trends of improvement of IQ scores with increased IAK and reduction of most IQ scores with increased kVp were observed mostly with the IAEA phantom, but not with the commercial phantoms (for both automatic and visual scoring methods). For all phantoms, the maximum variations in IQ scores observed for repeated identical exposures were almost always below 10% with automatic evaluation whereas, for visual evaluation, reached 17%.

**Conclusions:**

Failure to detect some expected trends with the complex commercial phantoms may be attributed to the fact that IQ in DR is more strongly affected by the post‐processing procedures, which may mask the effect of other parameters on IQ, something that was not observed with the simple IAEA phantom.

## 1 INTRODUCTION

Digital radiography (DR) offers many advantages compared to screen‐film (SF) imaging. Its better dynamic range, higher resolution (in some situations), the automatically adjustment of images to accentuate the anatomical characteristics and facilitate clinical diagnosis, the image storage and image transfer capabilities, the abolishment of chemical developers, and reduction of costs are the main reasons why DR has almost entirely displaced SF imaging in more resourced countries, despite the possible higher costs of the initial investment.^1^, ^2^, ^3^, ^4^

DR has introduced some implications on traditional quality control (QC) testing of X‐ray modalities, including the evaluation of image quality (IQ). With SF radiography, the IQ of any acquired image could not be improved, as the optical density, contrast, and latitude of any image could not be modified after film development and fixing. The only possibility to enhance the visibility of various anatomical structures in an X‐ray film was to adjust viewing conditions (film viewer and ambient light illumination levels).^1^, ^5^ With DR, images can be processed in various ways to adjust and enhance the spatial resolution and contrast, and different post‐processing protocols applied onto the same raw image may result in different IQ levels.

For evaluation of IQ of DR images, physical phantoms like those used in SF imaging are still used. These phantoms contain various structures, and the ability of a human observer to detect these structures is used to quantify IQ. Various phantoms are commercially available, which commonly contain structures to assess spatial resolution (also referred to as high contrast resolution, HCR), low contrast (LC), and image latitude (which in DR is usually referred to as dynamic contrast). IQ metrics, such as signal‐to‐noise ratio (SNR) and contrast‐to‐noise ratio, which is also referred to as signal difference‐to‐noise ratio (SDNR), are also used to quantitatively assess IQ.^6^ However, the correlation of the previous metrics with clinical IQ is not straightforward and has been recently disputed.^7^ Recent literature suggests that IQ is best evaluated using modulation transfer function (MTF) for the evaluation of spatial resolution and detectability index (*d*′) for the evaluation of LC resolution (*d*′ depends on noise power spectrum and task‐transfer function).^7^ However, performance levels for acceptance or commissioning purposes, as well as for conformance to national or international norms, are still given in terms of lp/mm for HCR and in terms of visual detection of LC structures that have a specific nominal contrast difference with respect to the background.^5^, ^8^, ^9^, ^10^ Therefore, conformance must be confirmed using phantoms that can produce these metrics.

In DR, evaluation of IQ can be done visually as with SF images. Unfortunately, due to intra‐ and interobserver variability, scoring procedures based on visual observation are often subjective.^7^ To overcome this problem, some commercial phantoms are now accompanied with software for an automatic evaluation of IQ, to ensure that a certain DR image will be always scored exactly in the same way, irrespectively of how many times the evaluation is repeated. However, the cost of these phantoms and the accompanying software is considerable. Thus, medical physics departments usually buy only one phantom, and less often the corresponding software, for performing the required IQ tests in all radiography units under their supervision. This means that the possibility of daily or weekly IQ tests, especially for distant facilities, is practically negligible.

To facilitate IQ testing in DR (and allow for an increase in its frequency), IAEA recently developed a remote and automated solution using a simple, inexpensive phantom, and free software.^11^ The IAEA publication is accompanied by supplementary material to support the remote/automated QC process, including (1) real size blueprint of the proposed phantom allowing the user to accurately manufacture it, (2) dedicated free software called Automated Tool for Image Analysis (ATIA) to automatically analyze the phantom images and provide advanced and sophisticated metrics of IQ (exported in excel file format), (3) an MS Excel file where the IQ results from each test can easily be fed, for proper documentation of the results and creation of long‐time performance charts for each radiographic facility, (4) a user manual of the ATIA software.^12^ The IAEA methodology was tested in a pilot study in various clinical scenarios, and initial results showed that the phantom is easily fabricated and enables QC tests to be performed even on a daily or weekly basis, as the software allows for a complete and automated evaluation of the principal performance characteristics of the imaging chain.^13^ To test the IAEA methodology in wide clinical scenarios, the IAEA launched in 2021 a Coordinated Research Project (CRP) entitled “Advanced Tools for Quality and Dosimetry of Digital Imaging in Radiology” (E24025).^14^

The main objective of this work was to evaluate IQ metrics obtained using commercially available solutions (phantoms and software) and the IAEA radiographic phantom solution. Apart from evaluating similarities and/or differences among phantoms in terms of IQ metrics, the most important question to be answered is whether these phantoms and the related software are sensitive enough to detect subtle changes in IQ. These changes may occur during QC tests performed on different dates for the same radiography unit, due to fluctuations in the exposure factors typically used to acquire the phantom images, as a result of a malfunction or a change in the system's adjustments.

## 2 MATERIALS AND METHODS

**FIGURE 1:**
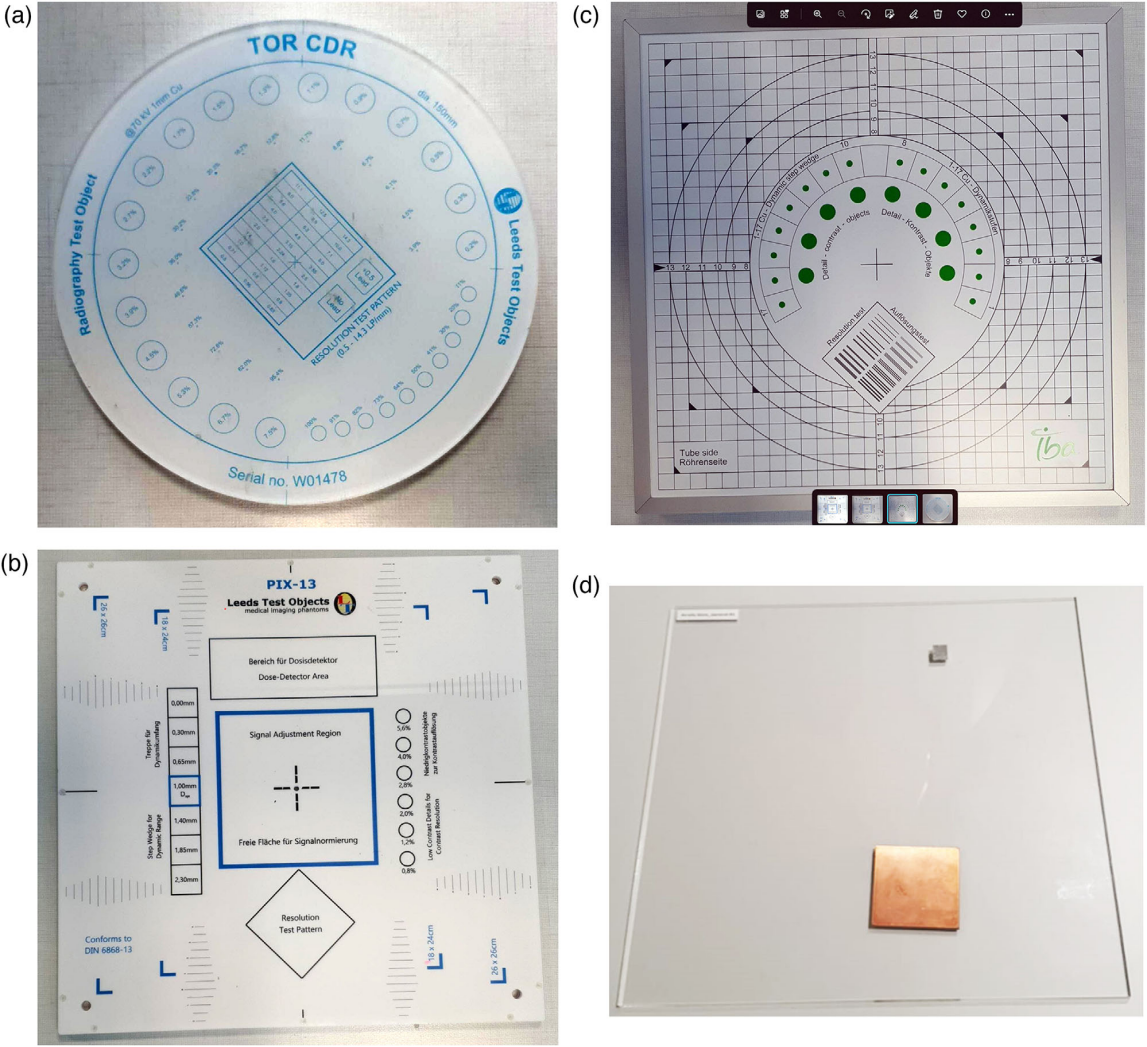
Photographs of the commercial phantom and the IAEA phantom: (a) Leeds TOR CDR, (b) Leeds PIX‐13, (c) IBA Primus A, (d) IAEA radiographic phantom

**FIGURE 2:**
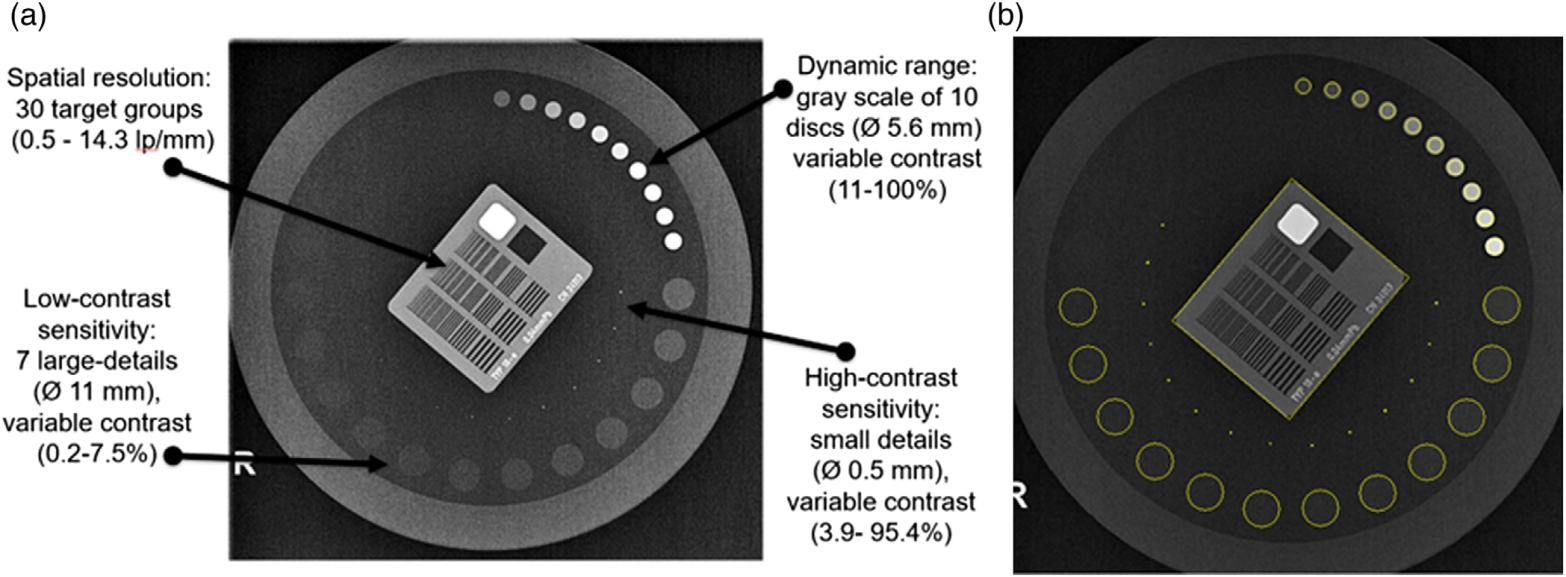
(a) Characteristics of the Leeds TOR CDR phantom (5 mm polymethylmethacrylate [PMMA] plus 1 mm Cu positioned on the X‐ray tube collimator), (b) auto‐recognition and delineation of targets and details of the phantom by the software

**FIGURE 3:**
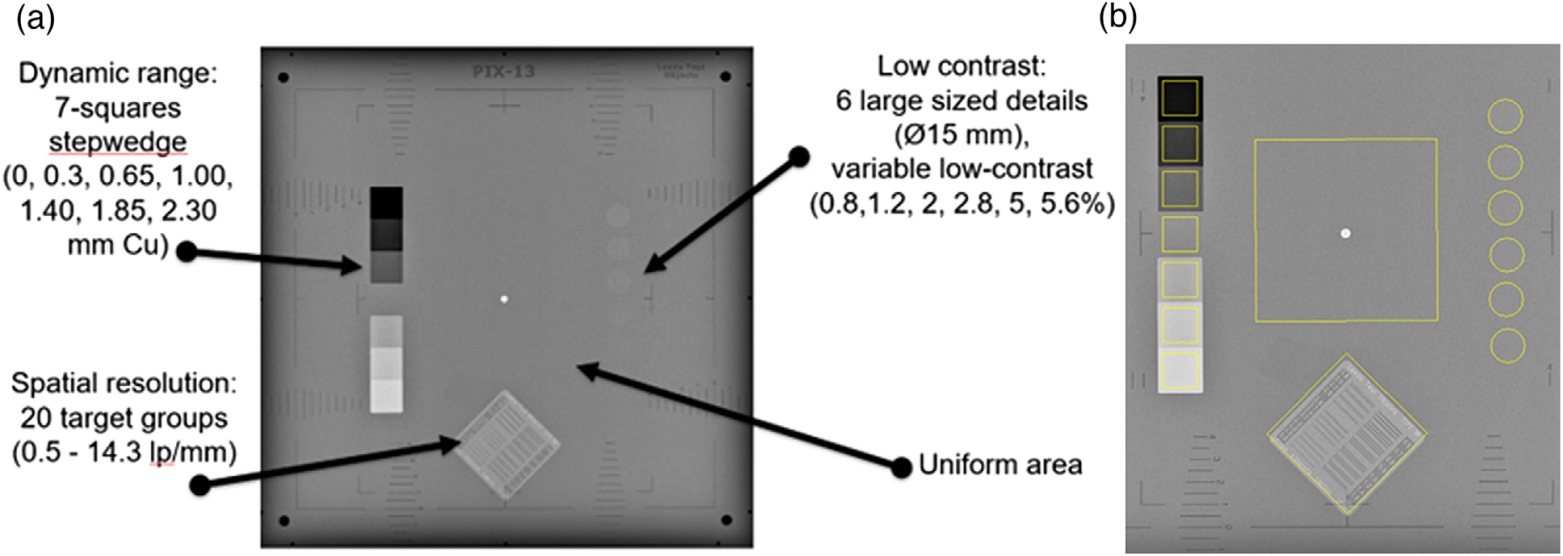
(a) Characteristics of the Leeds PIX‐13 phantom (8 mm polymethylmethacrylate [PMMA] plus 1 mm Cu positioned on the X‐ray tube collimator), (b) auto‐recognition and delineation of targets and details of the phantom by the software

**FIGURE 4:**
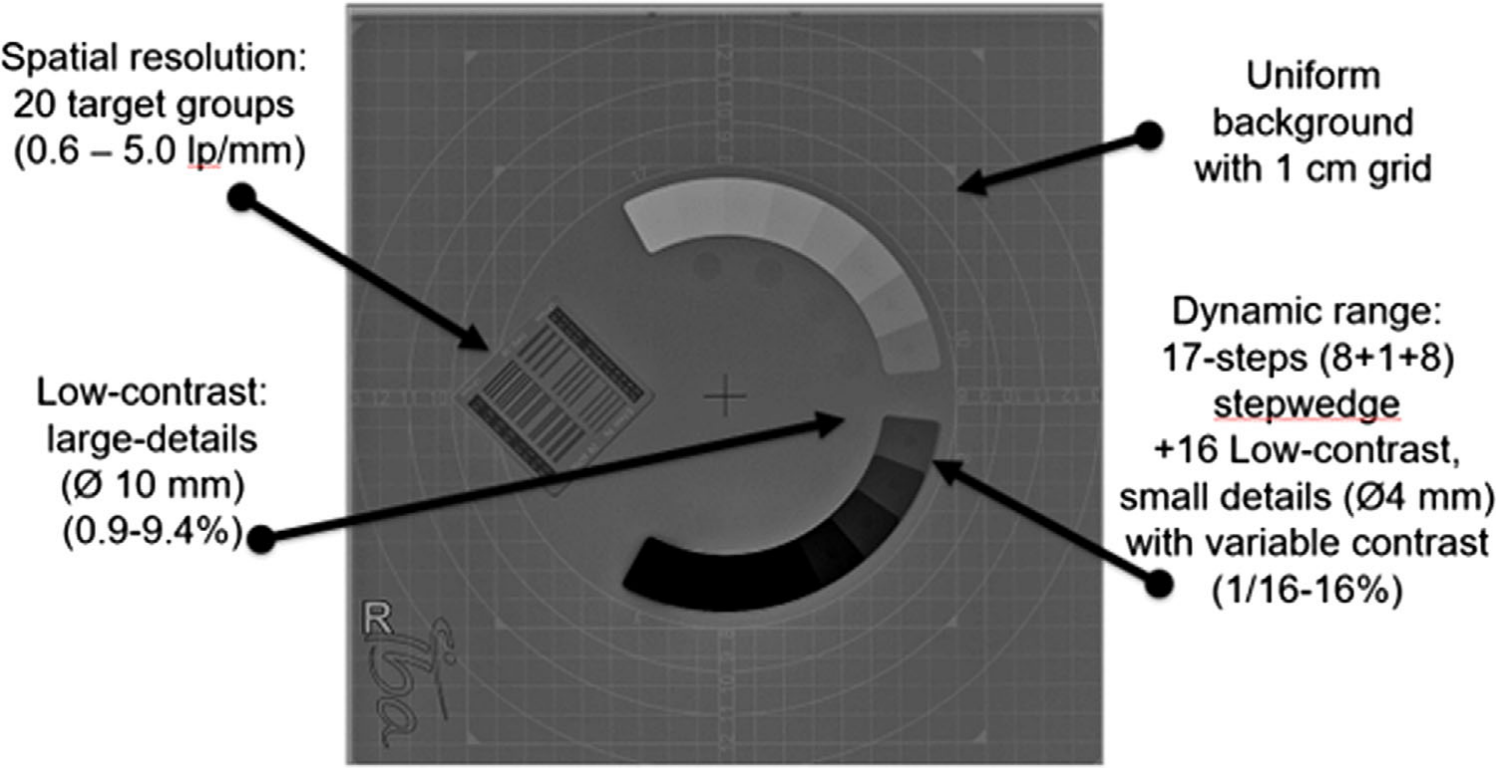
Characteristics of the IBA Primus A phantom (17 mm polymethylmethacrylate [PMMA] plus 1.5 mm Cu incorporated in the phantom)

**FIGURE 5:**
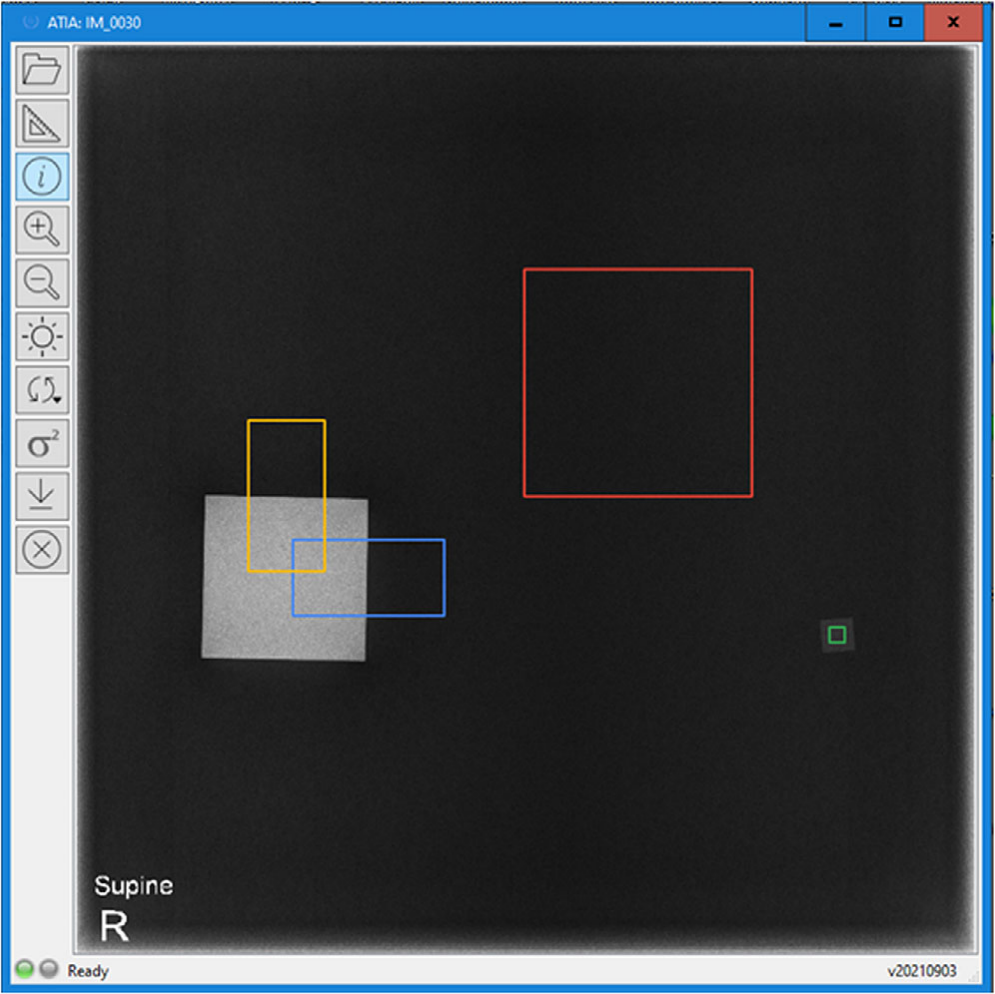
Radiographic appearance of the IAEA phantom within the Automated Tool for Image Analysis (ATIA) software. The regions of interest (ROIs) automatically positioned by the software to calculate the image quality (IQ) metrics (orange and blue for modulation transfer function [MTF], red and green for signal difference‐to‐noise ratio [SDNR], signal‐to‐noise ratio [SNR], and *d*′) are shown. It must be noted that the phantom is accompanied by a 2 mm Cu sheet which is positioned in the collimator, to simulate the attenuation of an average patient.

Four phantoms were used in this study (Table 1 and Figure 1), including three commercial (Leeds TOR CDR, Leeds PIX‐13, and IBA Primus A) and one henceforth referred to as the IAEA phantom, locally manufactured (made of PMMA, Cu, and Al) according to IAEA specifications and guidance described in IAEA CRP E24025 project‐related documentation.^11^, ^13^ The characteristics of the four phantoms and the corresponding IQ metrics calculated automatically from each phantom (using the respective software) are shown in Figures 2, 3, 4, 5, for CDR, PIX‐13, Primus, and IAEA phantom, respectively, and summarized in Table 1.

**TABLE 1:** Main image quality (IQ) metrics reported by the dedicated software and other relevant information

Phantom	Software	Spatial resolution	Low‐contrast sensitivity	Other	Dynamic range
CDR	AutoPIA (CyberQual)	MTF @ cutoff, 50%, 20%, and 10%	Number of visible large details	High‐contrast sensitivity: number of visible small sized details	Analysis of each one of the discs (PV, PVSD, nominal contrast)^a^
PIX‐13	AutoPIA (CyberQual)	MTF @ cutoff, 50%, 20%, and 5%	Number of visible large details	Homogeneity	Analysis of each one of the squares (PV, PVSD, relative contrast)
Primus	IQ Analyzer Primus (IBA)	MTF curves^b^	SNR (%)^c^	Uniformity, geometrical distortion	Dynamic scale: PV versus step‐wedge number^c^
IAEA	ATIA	MTF @50%, 20%, and 10%	SNR, SDNR, detectability (*d*′) for 0.3 and 4 mm diameter circular details^d^	–	–

Abbreviations: ATIA, Automated Tool for Image Analysis; MTF, modulation transfer function; SDNR, signal difference‐to‐noise ratio; SNR, signal‐to‐noise ratio.

^a^ PV: pixel value; PVSD: pixel value standard deviation. As all 10 details were visible at all images, this metric was not considered.

^b^ The MTF20% was calculated by interpolation.

^c^ The number of visible large low‐contrast details in the background, the number of visible step‐wedge steps and the number of visible small details within the step wedge cannot be determined by the software. The reported SNR (%) values are equal to the ratios of PVSD and PV of regions of interest (ROIs) positioned in the background area of the phantom multiplied by 100. Therefore, they are noise‐to‐signal ratio values (NSR) rather than SNR.

^d^ There are no circular details in the phantom. The detectability index (*d*′) is estimated from the noise power spectrum (NPS) and task‐transfer function (TTF) for specific object task, which in this case is the detection of circular details of the given diameters.^7^ The SNR is calculated as the ratio of the PV and the PVSD of the ROI in the background area of the image (the red ROI of Figure 5) and the SDNR as the difference of the PVs of the ROIs in the aluminum (the green ROI in Figure 5) and the background ROI, divided by the PVSD of the background ROI.

**TABLE 2:** Summary of the image quality (IQ) evaluation results for all four phantoms

	Phantom		CDR		PIX‐13		Primus		IAEA	
	IQ metric		Auto	Visual	Auto	Visual	Auto	Visual	Software	
**1. Repeated acquisitions (Poisson statistics)**	Spatial resolution: MTF20% or HCR (visual)	Min	3.10	3.28	3.10	2.90	4.83	2.70	4.22	***4.83***
		Max	3.31	3.42	3.39	3.40	5.21	3.20	4.40	***5.12***
		Mean	3.21	3.38	3.23	3.13	4.99	3.02	4.27	***4.91***
	Low‐contrast sensitivity or *SNR* or ***SDNR***	Min	12.0	11.7	6.0	5.0	*3.14*	6.3	*28.8*	***7.8***
		Max	13.0	12.3	6.0	5.7	*3.20*	7.3	*30.0*	***8.3***
		Mean	12.5	12.1	6.0	5.2	*3.17*	6.9	*29.5*	***8.0***
	Visibility 0.5 mm details or **# of dark, # of light steps** or *d (0.3 mm)* or ***d′ (4 mm)***	Min	14.0	11.3				**3.0, 4.0**	*3.18*	***53.0***
		Max	16.0	11.7				**3.7, 5.3**	*3.33*	***55.6***
		Mean	14.5	11.6				**3.4, 4.9**	*3.25*	***54.0***
**2. Increasing IAK on image receptor**	Spatial resolution: MTF20% or HCR (visual)	Min	2.93	3.17	3.02	2.80	3.14	2.70	3.93	***4.27***
		Max	3.35	3.70	3.67	3.40	5.33	3.50	4.82	***5.63***
		Mean	3.18	3.38	3.27	3.10	4.60	3.22	4.38	***4.78***
		Trend	–	0.44	–	0.35	–	–	0.33	***0.43***
	Low‐contrast sensitivity or *SNR* or ***SDNR***	Min	11.0	11.3	5.0	3.7	*1.72*	5.7	*26.0*	***6.1***
		Max	15.0	12.7	6.0	5.7	*3.81*	7.3	*41.0*	***11.1***
		Mean	12.8	12.0	5.8	4.6	*3.00*	6.4	*32.8*	***8.8***
		Trend	0.31	–	–	–	*0.91*	–	*−0.82*	***0.92***
	Visibility 0.5 mm details or **# of dark, # of light steps** or *d (0.3 mm)* or ***d′ (4 mm)***	Min	12.0	11.0				**2.7, 4.0**	2.55	***43.4***
		Max	17.0	12.7				**3.7, 5.3**	4.37	***72.9***
		Mean	14.3	11.8				**3.1, 5**	3.57	***59.9***
		Trend	–	–				**–, 0.34**	0.89	***0.88***
**3. Increasing kVp**	Spatial resolution: MTF20% or HCR (visual)	Min	2.73	3.17	2.25	2.90	3.51	2.70	4.09	***4.50***
		Max	3.31	3.85	4.48	3.40	6.08	3.60	4.50	***5.11***
		Mean	3.08	3.44	3.2	3.07	4.83	3.26	4.35	***4.91***
		Trend	–	−0.81	–	−0.34	–	–	–	***0.40***
	Low‐contrast sensitivity or *SNR* or ***SDNR***	Min	11.0	11.0	5.0	4.0	*2.98*	5.3	*29.2*	***5.7***
		Max	16.0	13.7	6.0	5.7	*3.52*	7.3	*31.5*	***10.3***
		Mean	13.2	12.2	5.7	4.9	*3.24*	6.0	*30.4*	***7.9***
		Trend	−0.39	−0.81	−0.56	−0.63	*0.6*	–	*0.88*	***−0.94***
	Visibility 0.5 mm details or **# of dark, # of light steps** or *d (0.3 mm)* or ***d′ (4 mm)***	Min	12.0	10.3				**2.3, 3.3**	*2.32*	***39.2***
		Max	17.0	14.7				**5.7, 7**	*4.08*	***68.1***
		Mean	14.9	12.1				**4.3, 5.5**	*3.24*	***54.1***
		Trend	−0.67	−0.82				**0.94, 0.89**	*−0.98*	***−0.97***
**4. Increasing additional filtration**	Spatial resolution: MTF20% or HCR (visual)	Min	2.95	3.28	3.10	2.90	4.27	2.70	4.09	***4.72***
		Max	3.31	3.57	3.51	3.40	6.02	3.60	4.36	***5.10***
		Mean	3.14	3.43	3.26	3.17	4.98	3.29	4.25	***4.88***
		Trend	–	–	–	–	–	0.50	−0.55	***−0.56***
	Low‐contrast sensitivity or *SNR* or ***SDNR***	Min	12.0	11.3	5.0	5.0	*3.14*	6.0	*28.9*	***8.1***
		Max	15.0	12.3	6.0	5.7	*3.35*	7.3	*29.6*	***8.3***
		Mean	12.9	12.0	5.8	5.1	*3.25*	6.5	*29.3*	***8.2***
		Trend	–	–	–	–	*0.82*	0.50	*–*	***0.94***
	Visibility 0.5 mm details or **# of dark, # of light steps** or *d (0.3 mm)* or ***d′ (4 mm)***	Min	12.0	11.3				**2.7, 4.0**	*3.21*	***52.9***
		Max	17.0	12.7				**3.7, 5.7**	*3.33*	***55.7***
		Mean	14.5	12.0				**3.0, 5.1**	*3.26*	***54.0***
		Trend	–	–				**–, 0.31**	*0.4*	***0.41***
**5. Changing post‐processing algorithm**	Spatial resolution: MTF20% or HCR (visual)	Min	2.80	3.42			3.84	3.00	2.47	***2.73***
		Max	4.01	3.55			5.30	3.30	4.32	***5.11***
		Mean	3.39	3.53			4.60	3.19	3.62	***4.00***
		Trend	+	ø			+	ø	+	***+***
	Low‐contrast sensitivity or *SNR* or ***SDNR***	Min	12.0	10.7			3.14	5.7	*20.3*	***6.0***
		Max	14.0	12.3			5.14	6.7	*40.6*	***12.5***
		Mean	12.6	11.4			3.61	6.4	*28.9*	***10.0***
		Trend	+	+			*+*	+	*+*	***+***
	Visibility 0.5 mm details or **# of dark, # of light steps** or *d (0.3 mm)* or ***d′ (4 mm)***	Min	14.0	11.3				**2.7, 4.3**	*2.64*	***42.9***
		Max	17.0	12.7				**4, 5.7**	*4.62*	***79.5***
		Mean	14.3	11.8				**3.3, 5.1**	*3.73*	***64.7***
		Trend	+	+				**+**	*+*	***+***

*Note*: Trends with *R* ^2^ < 0.3 are denoted with a dash. The symbol in front of the *R* ^2^ value denotes a negative trend. For nonnumerical values of *X*‐axis (i.e., acquisition protocols), intense variations are denoted in the trend row with “+”, whereas “ø” denotes minor variations. Bold, italics, and bold italics characters are used to denote different metrics and respective score values reported in the same table row. For the IAEA phantom, two MTF20% values are reported: the horizontal (normal font) and the vertical (bold italics). The “+” denotes variation (≥10%), whereas “ø” denotes no variation (<10%).

Abbreviations: HCR, high contrast resolution; IAK, incident air kerma; SDNR, signal difference‐to‐noise ratio; SNR, signal‐to‐noise ratio.

The X‐ray unit used to acquire the phantom images was a Luminos dRF Max (Siemens, Healthineers, Munich, Germany), which has a digital flat panel detector for both fluoroscopy and radiography. This unit has been subjected to an extensive QC procedure prior to the following experiments, and all parameters, including kVp accuracy, and kVp, incident air kerma (IAK), and Automatic Exposure Control (AEC) system repeatability, were well within the adopted performance limits. To investigate the effect of the exposure factors on the IQ scores, the four phantoms were sequentially positioned on the radiographic table, and the following acquisitions were made:

1. Repeated acquisitions using the basic acquisition protocol as follows: abdomen examination protocol, tube potential of 70 kV, AEC adjusted for an IAK of 2.5 μGy on the image receptor, central AEC chamber activated, no additional filtration, and the grid in place. The choice of 70 kV is due to the relatively small thickness of the three commercial phantoms and to the fact that the nominal contrast values for CDR and PIX‐13 are reported for 70 kV, and for Primus for 75 kV. For the IAEA phantom, being the most attenuating of all phantoms due to the 2 mm Cu attenuator, the tube potential of the basic protocol was set at 81 kV as suggested by IAEA.^11^, ^13^ Any observed variation in IQ scores of the DR images resulting from repeated acquisitions should mainly be attributed to Poisson statistics, and to normal minor variations occurring in exposure factors during repeated exposures under AEC with this radiographic system.
2. Acquisitions with all four phantoms using the basic Abdomen protocol, except that IAK on the image receptor was varied (using the AEC dose level corrections −2, −1, +1, and +2 and direct IAK adjustment for AEC at 1.25 and 5 μGy). The objective of this experiment was to investigate whether IQ scores increase with IAK.
3. Acquisitions using the basic Abdomen protocol, except that kV was varied. In addition to 70 or 81 kV used, respectively, for the basic protocol for the three commercial phantoms and the IAEA phantom, other kVps in the range of 50–125 kV were selected (depending on the phantom thickness), to investigate whether IQ scores decrease with increasing kVp.
4. Acquisitions using the basic Abdomen protocol, except that additional filtrations of 0.1‐, 0.2‐, and 0.3‐mm Cu were used, to investigate whether IQ decreases with the use of harder beam qualities.
5. For CDR and Primus phantoms only, different images were produced from the same acquisition (reprocessing) using all the available selections of Diamond View (a post‐processing algorithm used in Siemens DR systems for optimizing IQ of different anatomic regions). For the IAEA phantom, various examination protocols were used to acquire different images as well. The aim was to investigate the effect of different protocols (i.e., post‐processing algorithms) on IQ scores.

Regarding visual evaluation, three human observers, with more than 5 years of experience in IQ scoring using QC phantoms, scored independently the radiographic images of the three commercial phantoms. The observers were blinded to the exposure factors and conditions used to acquire the images. The average value of the three readings was taken as the final score. The interobserver agreement was assessed using the Cohen Kappa statistics.

## 3 RESULTS

The main results of this study are tabulated in Table 2 (blocks 1–5).

**FIGURE 11:**
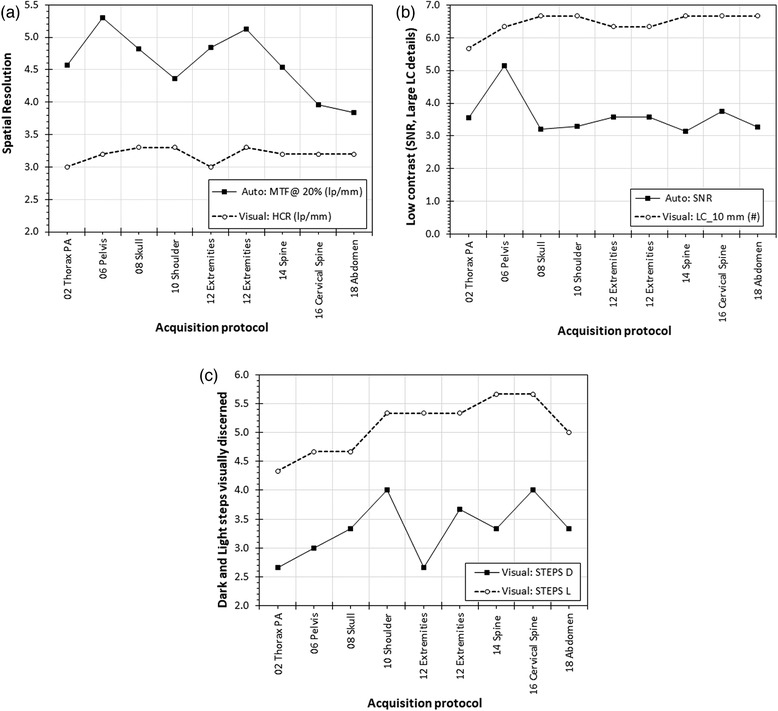
(a) Primus phantom: the effect of post‐processing algorithm on auto and visual evaluation of spatial resolution; (b) Primus phantom: the effect of post‐processing algorithm on auto evaluation of signal‐to‐noise ratio (SNR) and visual evaluation of low‐contrast details; (c) Primus phantom: the effect of post‐processing algorithm on visual evaluation of number of visible dark (D) and light (L) step‐wedge steps

**FIGURE 12:**
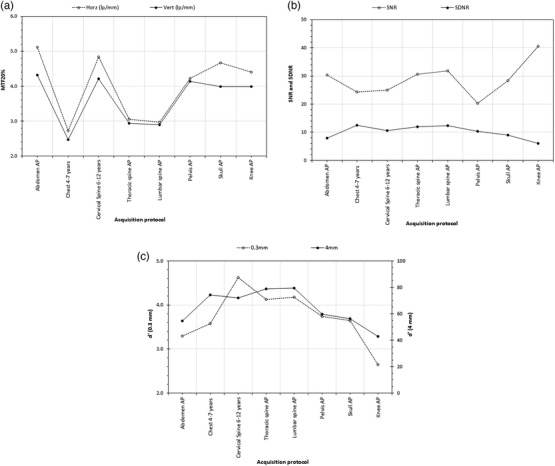
(a) IAEA phantom: the effect of post‐processing algorithm on automatic evaluations of horizontal and vertical spatial resolution; (b) IAEA phantom: the effect of post‐processing algorithm on automatic evaluations of signal‐to‐noise ratio (SNR) and signal difference‐to‐noise ratio (SDNR); (c) IAEA phantom: the effect of post‐processing algorithm on automatic evaluations of detectability

1. Regarding the maximum variation observed in IQ metrics with repeated identical exposures, the following observations were made:
  - The variation in mAs using AEC was minimal (∼1%) for all four phantoms, when keeping all exposure and geometrical parameters constant. During the performance testing of the X‐ray system, the excellent repeatability of kV (within 1%) had been also confirmed. It was thus deduced that variations in IQ values observed would be most likely due to Poisson statistics.
  - The maximum variations observed in the automatically calculated spatial frequency (MTF20%) values were less than 10% (range 6%–9%) for all three commercial phantoms. For the IAEA phantom, variations in horizontal and vertical MTF20% were 4% and 6%, respectively. Variations in the visual evaluations of the limiting spatial resolution (HCR) were 4% for CDR, 16% for PIX‐13, and 17% for Primus. It is important to note that for Primus, the MTF20% values were higher than the HCR values, whereas MTF20% and HCR values for CDR and PIX‐13 were all similar to each other and to HCR values from Primus. The MTF20% values calculated with the IAEA phantom were somewhere in between, but closer to MTF20% for Primus.
  - Maximum variations in the automatically calculated low‐contrast sensitivity and SNR values were 8% for CDR, 0% for PIX‐13, and 2% for Primus. For the IAEA phantom, variations in SNR and SDNR were 4% and 6%, respectively. For the visual evaluation of low‐contrast structures, the maximum variations were 6%, 13%, and 15%, respectively, for CDR, PIX‐13, and Primus.
  - The maximum variations in the scores of the automatic evaluation of small detail visibility with CDR were 14%, whereas for the visual evaluation, it was only 3%. The maximum variation in the number of visible dark and light steps in Primus reached 21% and 27%, respectively. For the IAEA phantom, the maximum variations in detectability values for the 0.3 and 4 mm were only 4% and 5%, respectively.
  - Overall, the IAEA phantom exhibited the smallest variation in IQ metrics, compared to the commercial phantoms.
2. The variations of the LC‐related IQ metrics for different IAK settings, calculated automatically or visually using the CDR, PIX‐13, Primus, and IAEA phantoms, are presented, respectively, in Figures 6, 7, 8, 9. The clinical exposure index, which is proportional to the IAK incident on the image receptor, is used as a surrogate quantity for IAK. These figures are typical examples of how the best fit lines to the data points (trend lines) were used to identify any possible variation pattern and report the positive or negative trend values given in Table 2. Regarding the effect of increasing IAK on the IQ, the following observations were made:
  - For the commercial phantoms, insignificant or weak trends with the increase of IAK were observed for all IQ metrics, except for Primus, where a logarithmic curve fitted almost perfectly the increase observed in SNR with IAK. However, it has to be reminded that the SNR values calculated for the Primus phantom are expressed as a percentage of the noise (pixel value standard deviation)‐to‐signal (pixel value) ratio (see the relevant footnote of Table 1), and therefore, it is expected that the observed trend would be the opposite of the respective SNR trend for the IAEA phantom.
  - For the IAEA phantom, an almost perfect linear increase with increasing IAK was observed for SDNR and detectability. On the contrary, for SNR, a clear trend of reduction with IAK increase was observed. For MTF20%, no clear trend was observed. The later should probably be expected, given the fact that for a given post‐processing protocol, spatial resolution is defined by the dexel size and thus should not be affected by IAK.
  **FIGURE 6:**
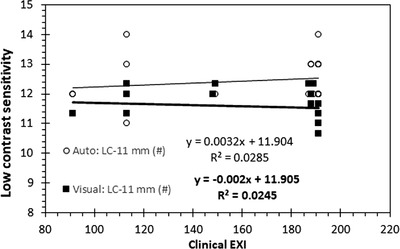
The effect of the incident air kerma (IAK) increase on automatic and visual scores related to low contrast is shown for the CDR phantom. (The clinical exposure index [EXI] is used as a surrogate quantity for IAK.)
  **FIGURE 7:**
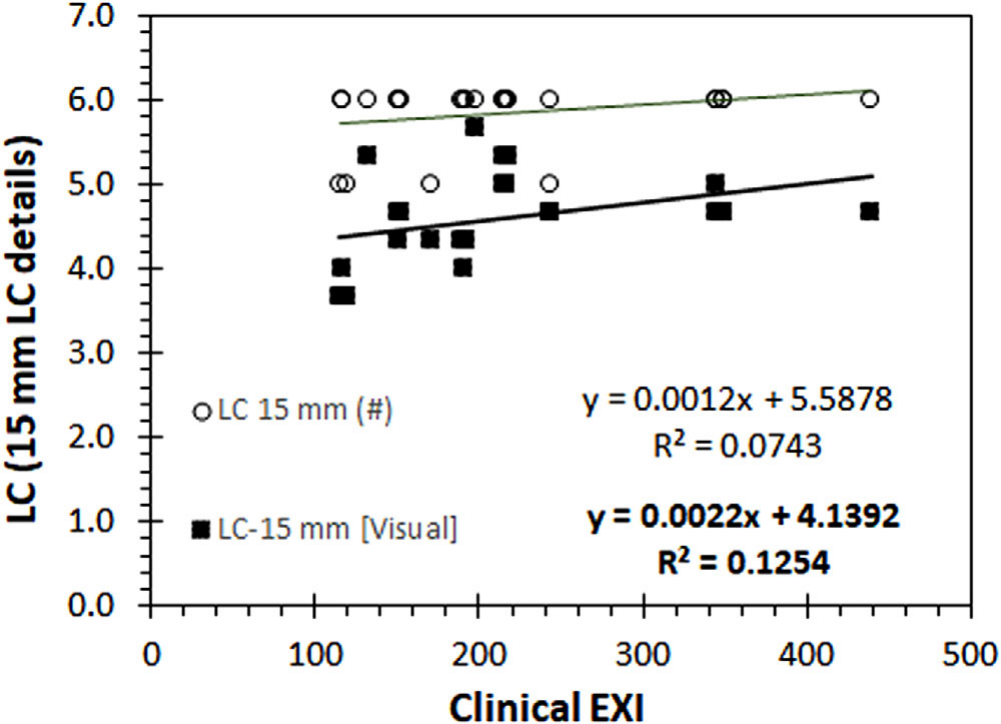
The effect of the incident air kerma (IAK) increase on automatic and visual scores related to low contrast is shown for the PIX‐13 phantom.
  **FIGURE 8:**
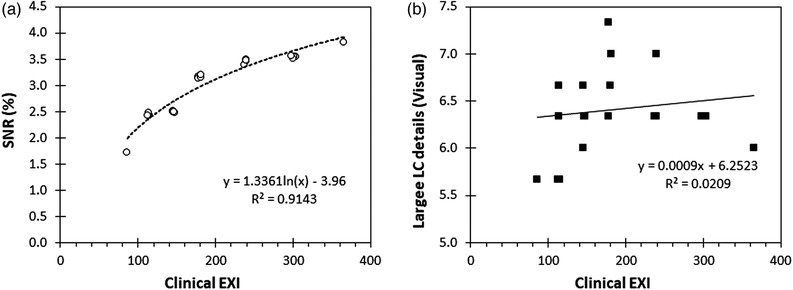
The effect of the incident air kerma (IAK) increase on automatic (a) and visual scores (b) related to low contrast is shown for the Primus phantom.
  **FIGURE 9:**
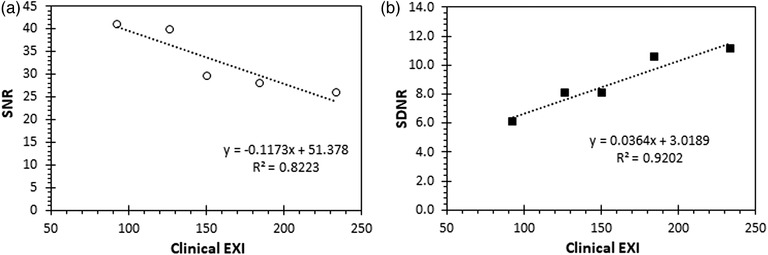
The effect of the incident air kerma (IAK) increase on signal‐to‐noise ratio (SNR) (a) and signal difference‐to‐noise ratio (SDNR) (b) is shown for the IAEA phantom.
3. Regarding the effect of increasing kVp on the IQ metrics, the following observations were made:
  - For all phantoms, the kVp increase did not seem to affect spatial resolution, except for CDR where HCR (visual evaluation) exhibited a negative trend.
  - For CDR, low‐contrast sensitivity (visual evaluation) and small detail visibility (automatic and visual evaluation) exhibited moderate‐to‐strong trends of reduction with kVp increase, whereas for the automatic evaluation of low‐contrast sensitivity, the negative trend was weak. Moderate trends of reduction of low‐contrast sensitivity with kVp increase were also observed for PIX‐13 (automatic and visual evaluation). For Primus, the SNR values exhibited a slight logarithmical increase with increasing kVp (the increase was numerically very small), whereas a strong trend of linear increase in the number of discernible dark and light steps with kVp increase was also observed. The latter may be attributed to the reduction of radiation contrast that occurs with the increase of kVp which may increase the range of different thicknesses that can be accommodated with any given post‐processing protocol.
  - For the IAEA phantom, a linear decrease was observed for SDNR and detectability with increasing kVp. In contrast, for SNR, an increase with increasing kVp was observed (as observed with IAK increase). This is indeed notable, as SNR seems to follow a different trend than SDNR and detectability.
4. Regarding the effect of increasing additional filtration on the IQ metrics, the following observations were made:
  - The increase of additional filtration produced small differences, but the trends in most cases ranged from very weak to moderate. Exception was the SNR for Primus, where a clear trend of logarithmical increase with increasing kVp was observed, but the increase was numerically very small (from 3.14 to 3.35).
  - For IAEA, a clear negative trend with increased filtration was observed only for SDNR.
5. Regarding the effect of post‐processing algorithm, as numerical values of trend cannot be defined, results are based on variations of IQ metrics on the graphs shown in Figure 10a–c for CDR, Figure 11a–c for Primus, and Figure 12a–c for the IAEA phantom. Thus, in Table 2, the “+” symbol denotes that a variation larger than 10% was observed, whereas the “ø” symbol denotes no variation (<10%). As can be seen in Figure 10a–c, some Diamond View settings are repeated once or twice. In the repetitions, a different post‐processing algorithm (usually attached to a respective examination protocol) setting was also used. Table 3 summarizes the effect of changing the post‐processing algorithm setting, and the symbols “+” and “ø” are used to denote variation (>10%) or not, in any of the IQ scores.
  **FIGURE 10:**
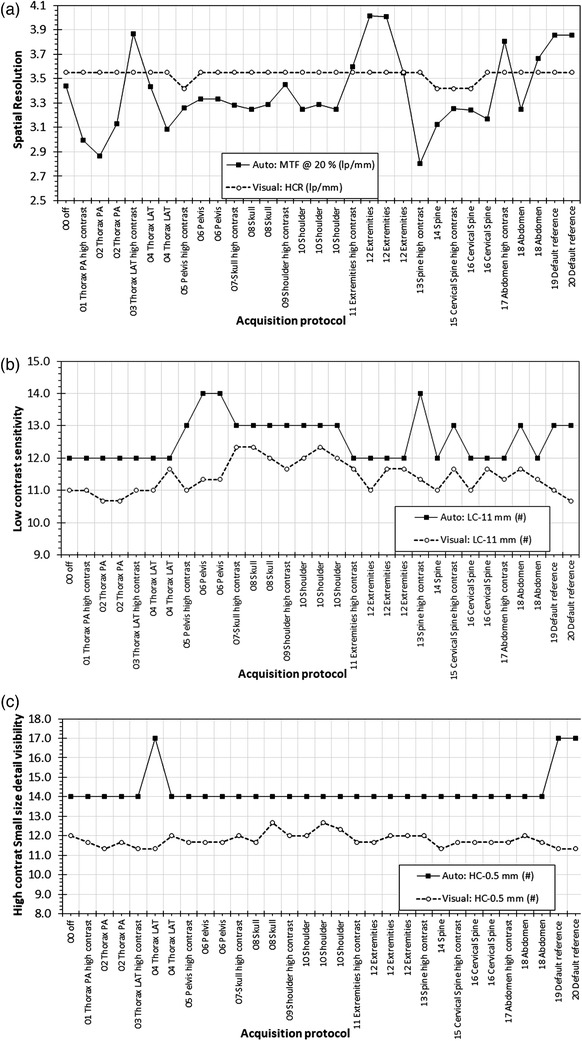
(a) CDR phantom: the effect of post‐processing algorithm on auto and visual evaluation of spatial resolution; (b) CDR phantom: the effect of post‐processing algorithm on auto and visual evaluation of low‐contrast sensitivity; (c) CDR phantom: the effect of post‐processing algorithm on auto and visual evaluation of high‐contrast sensitivity small sized details
  **TABLE 3:** Summary of variations observed in CDR phantom images, changing the post‐processing protocol

Acquisition protocol (number of images)	Auto: MTF @ 20% (lp/mm)	Visual: HCR (lp/mm)	Auto: LC‐11 mm (#)	Visual: LC‐11 mm (visual)	Auto: HC‐0.5 mm (#)	Visual: HC‐0.5 mm (visual)
02 Thorax PA (×2)	+	Ø	ø	Ø	ø	+
04 Thorax LAT (×2)	+	Ø	ø	+	+^a^	+
06 Pelvis (×2)	Ø	Ø	ø	Ø	ø	ø
08 Skull (×2)	+	Ø	ø	+	ø	+
10 Shoulder (×3)	+	Ø	ø	+	ø	+
12 Extremities (×3)	+	Ø	ø	+	ø	+
16 Cervical spine (×2)	+	+	ø	+	ø	ø
18 Abdomen (×2)	+	Ø	+	+	ø	+

*Note*: The “+” denotes variation, whereas “ø” denotes no variation.

Abbreviations: HCR, high contrast resolution; LC, low contrast; MTF, modulation transfer function.

^a^ Two values were observed (14 and 17), which cover the whole range of values observed with all the different post‐processing protocols tested.

As seen in Figure 10a, for CDR phantom, the automatically calculated MTF20% values presented intense variations with varying Diamond View settings. Differences were also observed even between images with the same Diamond View setting and different examination protocol setting (see also Table 3). On the contrary, variations in visual evaluation of spatial resolution were relatively small and did not follow the variations of MTF20%. As seen in Figure 10b, for low‐contrast sensitivity, variations between Diamond View settings were indeed observed in both automatic and visual evaluations and did not seem to follow a common pattern. However, variations in the automatic calculated values did not appear when changing the examination protocol setting (except for the “18 Abdomen” protocol). Finally, as seen in Figure 10c, for the detection of small sized/high contrast details, a value of 14 was reported from the software for all images, except for three protocols where 17 details were detected. It is notable, however, that in two repetitions with 04 Thorax LAT Diamond View setting protocol, the numbers of automatically detected low‐contrast details were 14 (Abdomen examination protocol) and 17 (Chest PA examination protocol). The respective visual evaluations presented small variations with different post‐processing algorithm settings.

For Primus, a small number of Diamond View settings were tested. As seen in Figure 11a,b, with different post‐protocols automatically calculated IQ metrics presented more intense variations, than those visually determined. However, in Figure 11c, it can be seen that the number of dark and light steps which are visually discerned also presented intense variations with post‐processing protocol changes. This verified that the dynamic range is affected by the selection of the post‐processing algorithm, something that was rather expected.

For the IAEA phantom, where a few images were acquired using different examination protocols instead of the reprocessing of the same image, as seen in Figure 12a–c, the different protocols affected all automatically calculated IQ metrics. The horizontal and vertical MTF20%, though different, followed similar variation pattern. On the contrary, variations of SNR and SDNR did not always follow the same pattern. The larger SNR value was observed for the Knee AP protocol, for which the smallest value of SDNR was observed; something is difficult to explain, but it is in‐line with the opposite trends noted before for SNR and SDNR with changing IAK and kVp. The detectability values estimated for small and large diameter details seemed to follow similar pattern (except for cervical spine 6–12 years protocol), though the small detail detectability was more intensely affected by protocol changes.

Finally, regarding the interobserver variability, the results of the Cohen Kappa statistics are presented in Table 4. It can be seen that except from one case, where the agreement was very good (PIX‐13: HCR, observers B and C), and two cases, where the agreement was good (CDR: HCR observers B and C; HC‐0.5 mm, observers A and C), in all other cases, the agreement between observers ranged from poor to moderate.

**TABLE 4:** Results of Cohen's Kappa^a^ statistics, between the image quality (IQ) scores of the three observers (A, B, and C), which performed the visual grading of the digital images

Phantom	IQ metric	No of valid cases	AB	AC	BC
CDR	HCR	79	0.49	0.52	0.75
	LC‐11 mm	79	0.18	0.46	0.06
	HC‐0.5 mm	79	0.23	0.63	0.25
PIX‐13	HCR	36	0.47	0.39	0.89
	LC	36	0.11	0.53	0.31
Primus	HCR	60	0.25	0.05	0.02
	LC	60	0.25	0.47	0.07
	Dark steps	60	0.13	0.07	0.45
	Light steps	60	0.32	0.10	0.05
Total	All	549	0.27	0.37	0.30

*Note*: Poor: 0.01–0.2, fair: 0.21–0.4, moderate: 0.41–0.60, good: 0.61–0.80, very good: 0.81–1.0.

Abbreviations: HCR, high contrast resolution; LC, low contrast.

^a^ Kappa value interpretation for agreement between reviewers’ scores.

## 4 DISCUSSION

The results of the Cohen Kappa statistics variability, shown in Table 4, are indicative of the basic problems inherent in the visual evaluation of IQ. Intra‐ but also interobserver variabilities are very well‐known problems regarding visual evaluations. The same observer may score differently the same image if he/she repeats the evaluation, another day or many months later, depending on whether he/she is tired or not, focused or in a hurry, and so on. In some cases, different observers may score differently the same image because they may have different visual acuities and/or follow different rules to define when a detail is considered visible or not. For example, regarding the last detail that may be considered half‐visible for two observers, one may decide to count it and the other not, and therefore scores will differ by 1. If the scoring of half‐points is allowed, it may reduce the difference in scores to half‐point or zero. Though score differences may be reduced using strict rules, they cannot be eliminated, and therefore, intra‐ and interobserver variabilities are always expected.

On the other hand, automatic objective evaluation of IQ is free of subjectivity, and results are expected to be always reproducible. However, IQ scores obtained using software may not be the same with the scores obtained from visual observation. As in the case of the CDMAM phantom, which is used for many years for the evaluation of IQ in mammography, conversion factors may be required to convert software scores to visual observer scores.^15^, ^16^, ^17^ Due to this reason, a direct comparison between software IQ scores and visual IQ scores was avoided. Furthermore, it should be also noted that in order to calculate an average score with the CDMAM phantom, it is required to acquire 8–16 images with the phantom slightly moved between acquisitions, because the relative position of the phantom's details (gold disks) and the dexels may affect the results. In the present study, to eliminate any variations that could be introduced by changes in position, each phantom was kept at the same position until all the images with different exposure conditions were acquired.

Currently, for the purposes of acceptance/commissioning and routing QC tests, the image is viewed usually on the monitor of the acquisition workstation, and the number of visible details is counted. The bigger the number of visible details is, the bigger the score and the better the IQ. The number of visible details is compared with relevant limits where applicable or with reference values established during commissioning. As image formation is affected by Poisson statistics, when IQ value is right on the limit, the Pass or Fail decision may change if the image acquisition is repeated. In this study, it was verified with all four phantoms that IQ may slightly change between subsequent acquisitions because of statistics.

One of the most important findings of this study was that for the three commercial phantoms, no well‐defined pattern of change in both automatic and visual IQ scores was observed with increasing IAK. This was rather unexpected, based on the general principle, that increase of IAK is expected to reduce noise and therefore increase IQ score. Although spatial resolution is mostly affected by dexel size and processing protocol and not by the IAK, for any given protocol, the detectability of details with various sizes and contrasts should be improved with the increase of IAK. In contrast to the commercial phantoms, for the IAEA phantom, the increase of IAK improved the SDNR and detectability values but had a rather unexpected negative effect on SNR.

Similarly, applying higher kVp, which is preferable from patient dose perspective, is expected to decrease radiation contrast and therefore the detectability. Such a clear trend was not observed with any of the commercial phantoms, regarding the IQ metrics automatically obtained, though in CDR a strong negative trend was observed in all visual scores. On the contrary, for Primus, a moderate positive trend in SNR with kVp increase was observed, which agreed with the stronger positive trend observed in the SNR values for the IAEA phantom with kVp increase. On the contrary, strong negative trends with kVp increase were indeed observed with the IAEA phantom for the SDNR and the detectability. Furthermore, the increase in the number of steps visually resolved with the increase of kVp using the Primus phantom was also noteworthy, though this could not be automatically detected by the respective software. It must be noted that the inability to determine automatically the number of discernible steps, the number of discernible small details within the step wedge, and the number of discernible large low‐contrast details in the background should be considered to be a major disadvantage of the software accompanying the Primus phantom.

The results of this study exhibited that the increase of additional filtration, which is also preferable from patient dose perspective, did not produce any strong negative trend in IQ in all four phantoms. This finding indicates that the use of additional filtration should be encouraged, to reduce the dose to the patient. The use of 0.1‐ and 0.2‐mm Cu reduces IAK by approximately 30% and 50%, respectively. However, this is something that needs further investigation and validation using clinical images.

Regarding the effect of the post‐processing algorithms incorporated in every acquisition protocol, it must be noted that these are optimized for clinical and not for phantom images and therefore for scoring IQ using phantoms, especially with the IAEA phantom, it may be more suitable if raw (for‐processing) images are used.^11^, ^13^ However, obtaining raw images is not always easy (depending on the X‐ray unit manufacturer, it may require access to the service mode); therefore, the second‐best option is to use the same examination protocol for all subsequent QC tests, to be comparable with previous results. In this study, a raw image of the IAEA phantom was obtained after removing all post‐processing from an image originally acquired with the Abdomen protocol. When this image was scored using ATIA, MTF was approximately halved, SDNR and detectability values did not significantly change, but SNR increased from 40 to about 1000. Though it would be interesting to investigate the effect of the exposure parameters on the raw images as well, this study was limited on the IQ of the images acquired using the clinical mode.

This study has some additional limitations as only four phantoms and one X‐ray unit were used. The results of this study cannot be generalized to other phantoms and their software, nor to other digital X‐ray units from other manufacturers, which may use image receptors of different technologies and different pre‐ and post‐processing algorithms. Another problem that was not addressed in this study and applies to visual evaluation only is the possible effect of the monitor screen on the scores (conventional computer screen vs. acquisition workstation monitor or vs. PACS workstation monitor). Finally, this study did not address the relevance of IQ scores obtained with phantom with the IQ of clinical images.

## 5 CONCLUSION

The study showed that the three commercial phantoms and their related software are not sensitive enough to detect subtle changes in IQ with automatic scoring methods, as they failed to exhibit a clear trend of IQ improvement with increased dose and IQ deterioration with increased tube potential. On the contrary, using the IAEA phantom and methodology, the scores regarding SDNR and detectability followed the trends, which were theoretically expected. However, regarding the opposite trends observed for SDNR and SNR with IAK and kVp, increase cannot be explained with certainty. It seems that the increase of transmission with kVp increases the signal and reduces the noise (i.e., the SNR), but the reduction of radiation contrast entails that the signal differences are getting smaller, and this is why SDNR and detectability reduce with kVp increase. As detectability is considered to be a more suitable IQ metric than SNR, the detectability and therefore the SDNR trend should be the correct one.

More work is needed to evaluate in more detail whether conventional QC phantoms are appropriate to be used with clinical examination protocols, and to what extent the results of evaluations with such phantoms (like the observed minimal effect of additional filtration on IQ) are applicable in clinical images. As aforementioned, these protocols are designed to accentuate minor anatomical differences, and the effect of dose and beam quality on IQ may be concealed by the superior effect of the processing protocol on IQ. What was made evident in this study is that the diversity of the post‐processing protocols and relevant adjustments is too large, and therefore, it is practically impossible to evaluate all of them, not even during the commissioning phase. Therefore, for long‐term monitoring of IQ, a single examination protocol used for a frequent clinical examination, like Abdomen AP or Chest PA, should be used.

## CONFLICT OF INTEREST

The authors have no conflict of interest to state.

## AUTHOR CONTRIBUTIONS

All authors substantially contributed to the conception or design of the research and analyzed/interpreted results. Moreover, all authors contributed to drafting the manuscript or revising it critically for important intellectual content.
